# Haemodynamics Regulate Fibronectin Assembly via PECAM

**DOI:** 10.1038/srep41223

**Published:** 2017-01-25

**Authors:** Zhongming Chen, Chris Givens, John S. Reader, Ellie Tzima

**Affiliations:** 1 Department of Cell Biology and Physiology, University of North Carolina-Chapel Hill, Chapel Hill, NC 27599, USA.; 2 Wellcome Trust Center for Human Genetics, Oxford OX3 7BN, UK

## Abstract

Fibronectin (FN) assembly and fibrillogenesis are critically important in both development and the adult organism, but their importance in vascular functions is not fully understood. Here we identify a novel pathway by which haemodynamic forces regulate FN assembly and fibrillogenesis during vascular remodelling. Induction of disturbed shear stress *in vivo* and *in vitro* resulted in complex FN fibril assembly that was dependent on the mechanosensor PECAM. Loss of PECAM also inhibited the cell-intrinsic ability to remodel FN. Gain- and loss-of-function experiments revealed that PECAM-dependent RhoA activation is required for FN assembly. Furthermore, PECAM^−/−^ mice exhibited reduced levels of active β1 integrin that were responsible for reduced RhoA activation and downstream FN assembly. These data identify a new pathway by which endothelial mechanotransduction regulates FN assembly and flow-mediated vascular remodelling.

The extracellular matrix (ECM) is critical for a host of physiological processes. Not simply a structural scaffold, the ECM is critical for a number of cellular functions, including proliferation and survival^1^,^2^,^3^. Specifically in the vasculature, the ECM plays an important role in capillary morphogenesis and vascular tube stabilization^4^,^5^,^6^. A major constituent of the ECM in many tissues is fibronectin (FN), which exists in both plasma and cellular forms^7^. FN is a glycoprotein consisting of multiple binding domains for a plethora of proteins, including integrins, fibrin, collagen, heparin and other FN molecules^7^. FN is indispensable for development and, interestingly, mice that lack FN expression are embryonic lethal due to cardiovascular defects^8^,^9^. FN also plays an important role during pathological processes: during early atherogenesis, FN deposition is promoted in areas of disturbed flow and is associated with endothelial dysfunction^10^,^11^,^12^,^13^.

FN monomers are soluble, and when assembled, form highly adhesive, insoluble fibrils. Polymerisation of FN into the ECM is important for both development and adult physiology. FN fibrillogenesis is essential for cleft formation during epithelial branching, the process by which nascent salivary glands begin developing^14^. FN fibrils also provide a mesodermal guidance cue during gastrulation^15^,^16^. Injection of an RGD peptide blocks FN fibrillogenesis in the amphibian blastocoel cell roof, leading to a loss of directionality in mesodermal migration^17^,^18^. In adult physiology, mutations in the Hep-II and Hep-III domains of FN are present in carriers of glomerulopathy with FN deposits (GFND)^19^. These mutations occur in regions of FN that are critical for fibrillogenesis, implicating impaired FN assembly in the progression of GFND. Additionally, FN fibrillogenesis regulates the remodeling and organization of other ECM molecules, as blocking FN polymerization also blocks collagen (CL) polymerization^20^. FN assembly is a complex process that involves FN binding with integrins, an increase in FN-FN interaction, and conversion to deoxycholate (DOC) insoluble FN fibrils^1^,^7^. The integrin binding step is primarily mediated by the FN binding integrin α5β1, which binds the RGD sites on FN molecules and increases clustering after binding occurs^7^,^21^. The small GTPase RhoA is also required for interaction of FN proteins. RhoA activity, and downstream activation of ROCK, increases cell contractility which acts upon FN proteins through α5β1 integrins, opening up cryptic binding sites in FN^22^. FN dimers assemble into fibrils that are eventually augmented to become large, DOC-insoluble FN polymers that make up the ECM. Although a great deal is known about the process of FN assembly, its importance in cardiovascular biology is incompletely understood. FN assembly is required for some endothelial functions, like network formation and proliferation^4^,^5^,^6^. Assembled FN also regulates phenotypic plasticity in smooth muscle cells^2^,^23^. Early studies suggested that ECs are involved in remodeling FN in the basal lamina and that FN organization, similar to actin stress fiber organization, shows flow dependency^24^,^25^. In the descending aorta, an area of the vasculature that experiences laminar flow, FN fibrils are thick and align in the direction of flow. However, at the renal artery bifurcation, an area of disturbed flow, FN fibrils are randomly distributed^25^. The role of FN assembly in vessel function was more definitively tested using a peptide that inhibits FN polymerization: when this peptide was utilised in a mouse model of partial carotid artery ligation (CAL), it inhibited intima-media thickening (IMT) and flow-mediated vascular remodeling^2^.

Vascular remodeling resulting from CAL is driven by haemodynamic forces, as CAL induces chronic disturbed flow in the ligated carotid artery^2^,^26^,^27^. Although it is appreciated that shear stress sensing by ECs is a major driving force for vascular remodeling, the molecular mechanisms by which endothelial mechanosignaling leads to vascular remodeling are unknown. We have previously shown that the cell adhesion molecule, PECAM, is a member of an endothelial mechanosensory complex. This complex is necessary and sufficient for endothelial mechanosignalling, including alignment of stress fibers in the direction of flow^28^. *In vivo*, PECAM is necessary for vascular remodeling after CAL^26^, as well as collateral remodelling in response to hindlimb ischaemia^29^. Using a magnetic tweezers system, we have shown that local force application on PECAM results in a global mechanotransduction response that involves remodeling of the actin cytoskeleton and activation of mechanical stiffening^30^. However, very little is known about the specific mechanisms underlying shear induced FN assembly. Here, we show that haemodynamics, and shear stress in particular, regulate FN assembly *in vitro* and *in vivo* in a PECAM-dependent manner. Mechanistically, PECAM-dependent FN assembly requires RhoA and β1 integrin activation. These data identify a new pathway by which endothelial mechanotransduction regulates FN assembly and flow-mediated vascular remodeling.

## Results

### *In vivo* flow-mediated FN remodeling is dependent on PECAM

**Figure 1:**
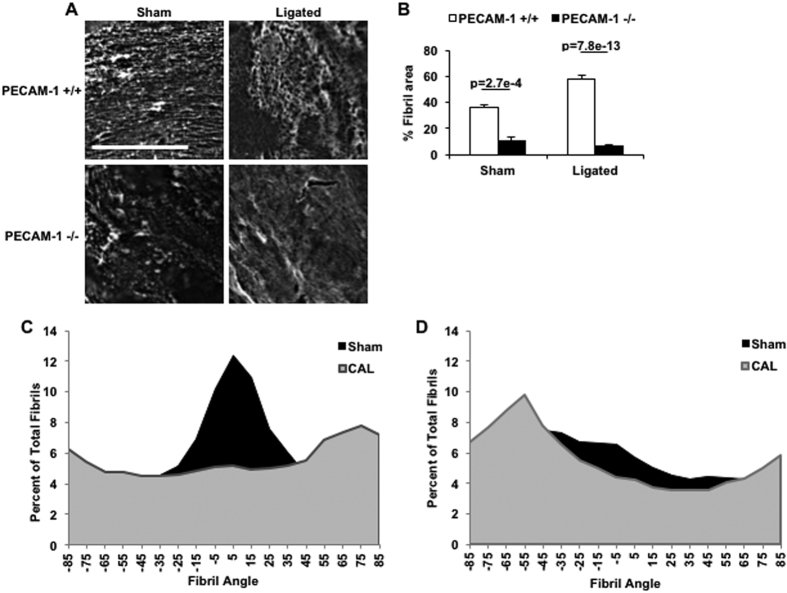
PECAM is required for FN assembly in response to CAL. (**A**) 3 weeks after CAL, sham and ligated carotid arteries were harvested, mounted *en face* and stained for FN. (**B**) FN fibril area was quantified. Scale Bar = 20 μm; n = 16 WT and 10 PECAM^−/−^ mice. (**C**) WT FN fibril angle was analyzed in sham and 3-week ligated LCAs n = 3 sham and n = 5 CAL. (**D**) KO FN fibril angle was analyzed in sham and 3-week ligated LCAs n = 2 sham and n = 3 CAL.

Previous work from our group has shown that PECAM^−/−^ mice exhibit impaired flow-mediated vascular remodeling and reduced IMT after partial CAL^26^. Since FN assembly is required for IMT^2^, we hypothesized that flow-mediated vascular remodeling, FN assembly and PECAM mechanotransduction are connected. To test this, we first examined if FN assembly is sensitive to changes in haemodynamics. We subjected WT mice to partial CAL or sham surgery, and after 3 weeks, left common carotid arteries (LCA) were mounted *en face* and immunostained with an anti-FN antibody. Sham LCAs, which experience laminar shear stress, exhibited long FN fibrils that were aligned in the direction of flow (Fig. 1A and C). In contrast, exposure of LCAs to low and disturbed flow due to ligation resulted in formation of thicker and randomly-oriented FN fibrils (Fig. 1A and C). Interestingly, LCAs from PECAM^−/−^ mice displayed differences in FN organization compared to WT LCAs in both sham and ligated conditions (Fig. 1A and D). Under laminar flow conditions present in sham LCAs, PECAM^−/−^ animals had few and randomly oriented FN fibrils; even after changing flow in response to ligation, PECAM^−/−^ LCAs failed to show the complex FN fibrils evident in the WT ligated LCAs, suggesting that absence of PECAM results in impaired flow-mediated FN assembly and remodeling (Fig. 1B). Consistent with the results in WT and PECAM^−/−^ carotid arteries, the WT descending aorta displays a high degree of FN assembly, whereas PECAM^−/−^ descending aortas lack complex FN assembly (Figure S1). Given that the FN matrix serves as a template for deposition of other ECM proteins^31^, we also examined the distribution of collagen I (CL) in sham and ligated LCAs. Similar to the observations made for FN fibrils, PECAM^−/−^ aortas were characterized by wispy and fewer CL fibrils (Figure S2A).

### Disturbed shear stress induces FN assembly in a PECAM-dependent manner

**Figure 2:**
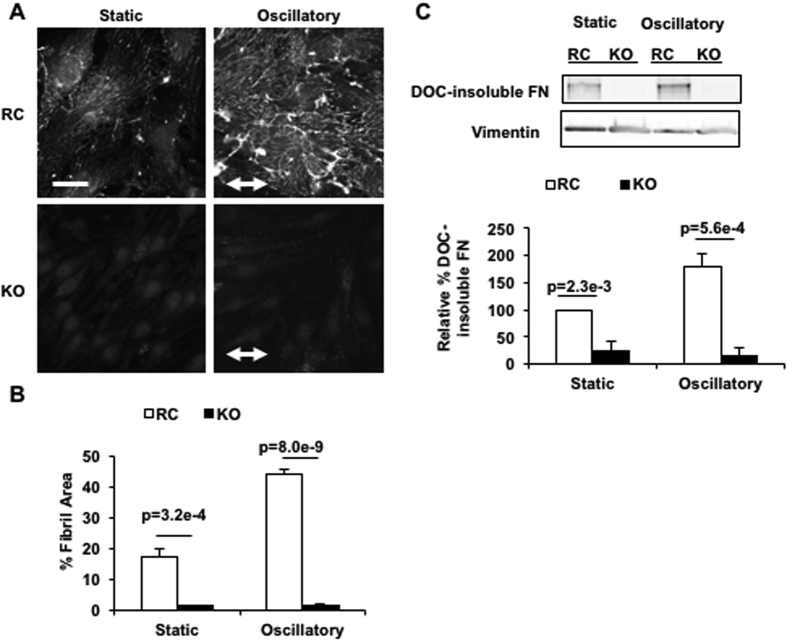
Disturbed shear stress induces PECAM-dependent FN assembly. RC and KO cells were subjected to flow or kept as static controls. (**A**) After flow, RC and KO cells were fixed and stained for FN, followed by (**B**) quantification of FN fibril area. (**C**) Representative western blots showing DOC-insoluble FN in RC and KO ECs before or after oscillatory shear stress. Quantification of DOC-insoluble FN relative to static RC lysates is shown, and was performed after normalization to vimentin. Scale bar in (A) is 20 um. (**A**,**B**) n = 5 independent experiments; (**C**) n = 4 independent experiments.

To determine whether the observed deficit in FN assembly in KO mice is an endothelial-specific phenotype, we exposed PECAM knockout (KO) and PECAM reconstituted (RC) ECs to oscillatory shear stress, followed by visualization of FN fibrils (Fig. 2A and B). Striking differences in FN assembly were observed between genotypes. RC cells showed a high degree of FN fibril formation in static conditions and responded to shear stress by increasing fibril formation 3-fold. In contrast, KO cells did not form any detectable FN fibrils in either static or sheared conditions. The few FN deposits that did form resembled the punctate nature of FN deposits observed in PECAM^−/−^ mice (Fig. 1). Similar results were obtained when ECs were immunostained for CL (Figure S2B). Interestingly, KO ECs also exhibited reduced FN mRNA levels, suggesting that PECAM is also important for FN expression (Figure S3).

FN assembly renders fibrils insoluble in DOC-containing lysis buffer, making DOC insolubility an appropriate biochemical method for assaying the degree of FN assembly. To complement our immunofluorescence data, we performed DOC-solubility experiments. RC and KO lysates were fractionated using a DOC-containing lysis buffer after application of shear stress, followed by immunoblotting for FN (Fig. 2C). In static conditions, RC cells showed 5-fold more assembled FN than KO cells. Application of shear increased the amount of assembled FN 2-fold in RC cells, while no increase in assembled FN was observed in sheared KO cells. These results are consistent with the immunofluorescence data and collectively show that PECAM is required for both basal and shear-dependent FN assembly and remodeling in ECs.

### PECAM is required for the intrinsic ability to assemble FN

**Figure 3:**
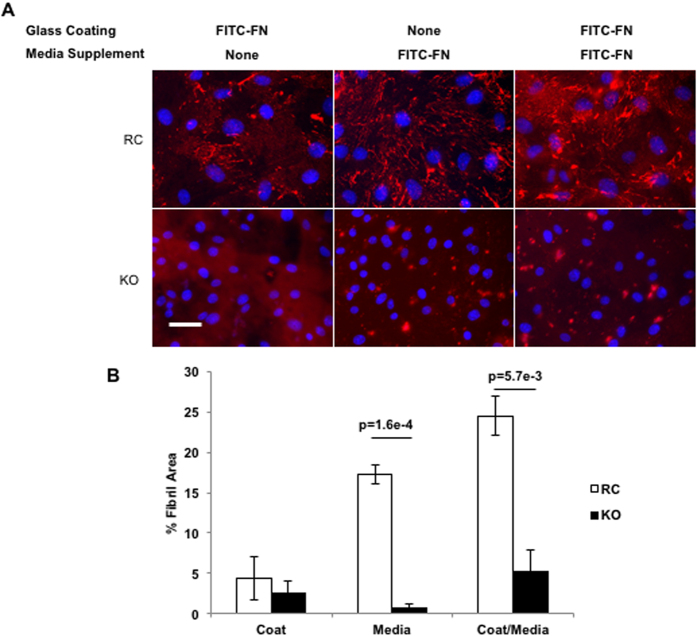
PECAM is required for the intrinsic ability of ECs to remodel FN. (**A**) FITC-conjugated FN was provided to static RC and KO cells as a tissue culture glass coat, media supplement, or both. Representative images are shown (FN was pseudocolored in red for clarity). Representative images are shown (FN was pseudocolored in red for clarity). Scale Bar = 40 μm (**B**) Percent fibril area was also quantified. (**A**,**B**) n = 3 independent experiments.

To determine whether PECAM is required for the cell-intrinsic ability to assemble FN, we utilized a fibrillogenesis assay whereby RC and KO cells were supplied with FITC-FN as ECM, soluble media supplement, or both, followed by fixation and fluorescence microscopy (Fig. 3). Under all three conditions, RC cells displayed increased accumulation of FITC-FN into fibrils compared to KO cells. Whether FITC-FN was provided as ECM glass coating or media supplement, RC cells assembled more extensive networks of FN fibrils. KO cells organized the FN into punctate deposits, but failed to form FN fibrils. When FN was provided as both a glass coating and a media supplement, RC cells demonstrated augmented assembly of fibrillar FN matrix. Under these same conditions, KO cells assembled significantly less FITC-FN-containing fibrillar matrix that was characterized by primarily punctate deposits of FN. Together, these data suggest that PECAM is required for EC fibrillar FN matrix assembly independent of the expression of endogenous FN.

### Disturbed shear stress regulates PECAM-dependent FN assembly via a β1-integrin-RhoA pathway

**Figure 4:**
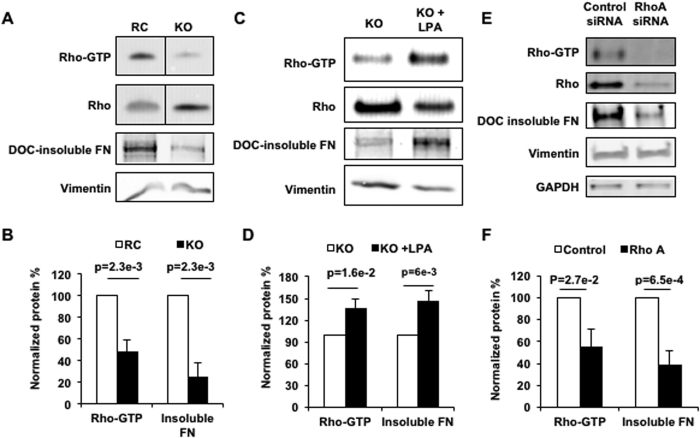
PECAM-dependent RhoA activity is required for FN assembly. (**A**) RhoA-GTP levels in RC and KO cells were determined by RBD pulldown. FN assembly was assayed by DOC-insolubility. (**B**) Quantification of RhoA-GTP and DOC-insoluble FN is below. (**C**) KO cells were stimulated with LPA, a RhoA agonist. RhoA-GTP was determined by RBD pulldown and FN assembly was assayed by DOC-insolubility. (**D**) Quantification of RhoA-GTP and DOC-insoluble FN is below. (**E**) ECs were transfected with control or RhoA siRNA and Rho-GTP levels and DOC-insuble FN were assayed. (**F**) Quantification of RhoA-GTP and DOC-insoluble FN is below. Experiments were performed in static cells. (**A**,**B**) n = 4 independent experiments; (**C**,**D**) n = 6 independent experiments; (**E**,**F**) n = 4 independent experiments.

FN assembly in fibroblasts requires actomyosin signaling via the Rho-ROCK pathway^7^,^22^,^32^. Notably, RhoA-dependent contractility is required for exposure of a cryptic self-assembly site in the FN protein^22^. Additionally, knockdown of ROCK isoforms I or II leads to reduced FN matrix assembly and altered MLC localization, further suggesting a role for RhoA-dependent contractility in FN assembly^33^. Since KO cells lack the ability to properly assemble FN into fibrils, we examined the role of the Rho pathway in our system. We assayed RhoA activation levels in RC and KO cells by RBD pulldown, which uses the Rho binding domain from the Rhotekin protein to bind and isolate GTP bound RhoA. As shown in Fig. 4A and B, KO ECs have reduced levels of active RhoA compared to RC cells. The observed reduction in Rho-GTP levels correlates with lower DOC-soluble FN in the KO cells. To determine whether RhoA activation levels were causative for FN assembly, we performed gain-and loss-of-function experiments. Firstly, to determine if low FN assembly levels could be rescued by stimulation of RhoA activity, we treated KO cells with lysophosphatidic acid (LPA) to activate RhoA^34^, followed by RBD pulldown and DOC insolubility assays (Fig. 4C and D). LPA stimulation increased Rho-GTP levels approximately 140%, as expected, which was also accompanied by a concomitant and comparable increase in FN assembly compared to unstimulated cells. RhoA knockdown experiments were also performed in RC cells to determine whether decreased expression of RhoA reduces levels of assembled FN (Fig. 4E and F). Indeed, Rho-GTP levels were significantly reduced after RhoA knockdown, leading to a 60% reduction in DOC-soluble FN levels. Overall, these gain-of-function and loss-of-function studies show that PECAM-dependent RhoA activation is necessary for FN assembly in ECs.

**Figure 5:**
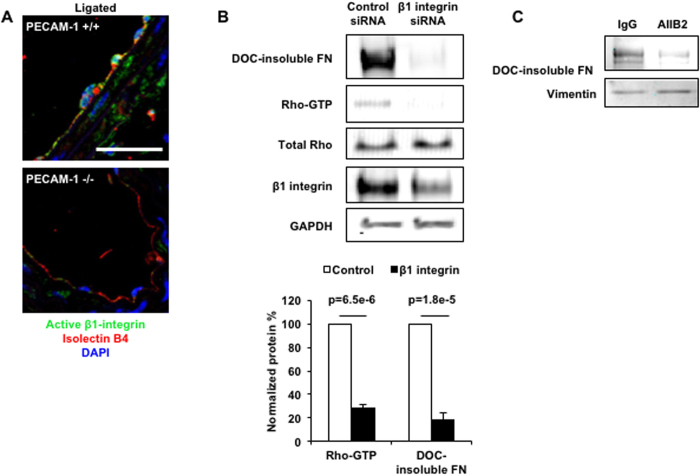
Role of β1 integrin activation in FN assembly. (**A**) Active β1-integrin staining in cross sections of WT and PECAM^−/−^ carotid arteries harvested 5 days post-CAL. Scale Bar = 20 μm; n = 3 independent experiments. (**B**) ECs were transfected with control or β1 integrin siRNA and levels of GTP-RhoA and DOC-insoluble FN were assayed and quantified. (**C**) ECs were incubated with control IgG or the β1 integrin function blocking antibody AIIB2 before determination of FN assembly by DOC-insolubility. (**B**) n = at least 3 independent experiments. (**C**) n = 4 independent experiments.

β1 integrin is a major FN binding integrin, and is reported to be the master integrin involved in stretching and assembling individual FN monomers into FN fibrils^21^. Given the deficit in FN assembly in the absence of PECAM, we examined the activation status of β1 integrins in the absence of PECAM. We performed immunofluorescence staining using an antibody specific for active β1 integrins in WT and PECAM^−/−^ carotid arteries. Imaging revealed a stark difference between the two genotypes (Fig. 5A). WT carotids displayed high levels of β1 integrin activation, concentrated in the endothelial layer, compared to PECAM^−/−^ carotids. These results raise the intriguing possibility that levels of active integrins are connected to both activation of RhoA and downstream FN assembly. To test this, we performed β1 integrin knockdown experiments (Fig. 5B). In RC cells treated with β1 integrin siRNA, Rho-GTP levels were reduced by 70% compared to control siRNA transfected ECs. This was accompanied by a comparable reduction in levels of DOC-insoluble FN, indicative of reduced FN assembly. In complementary experiments, when using a β1 integrin function-blocking antibody we also saw reduced levels of DOC-insoluble FN (Fig. 5C). Overall, these data show that PECAM-dependent β1 integrin activation is required for RhoA activation and downstream FN assembly in ECs.

## Discussion

**Figure 6:**
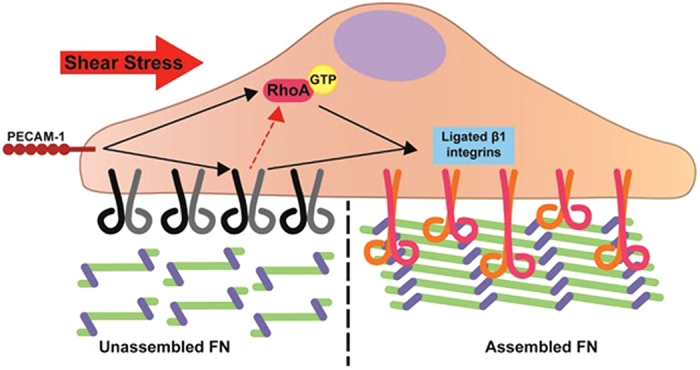
Distrubed flow regulates PECAM-dependent FN assembly through a β1-integrin-RhoA pathway. Distrubed flow regulates FN fibrillogenesis; mechanistically, this is dependent on PECAM-dependent activation of β1 integrins and RhoA.

We have previously shown that PECAM knockout mice exhibit impaired flow-mediated vascular remodeling and IMT in response to CAL^26^. Similarly, mice treated with a FN polymerization inhibitor are also characterized by reduced IMT and vascular remodeling^2^. Although endothelial responses to shear stress are known to regulate expression and deposition of ECM components^11^,^35^,^36^,^37^, virtually nothing is known about the role of shear stress in the regulation of FN assembly. We hypothesized that PECAM, a known endothelial mechanosensor is important for regulation of the organization of the subendothelial ECM, especially in response to shear stress. Our results here provide a mechanistic link between mechanosensing and FN organization during flow-mediated vascular remodeling. Using both PECAM knockout ECs and mice, we show that PECAM regulates FN fibrillogenesis in a RhoA- and integrin β1-dependent pathway (Fig. 6).

CAL is a method for modification of the carotid artery hemodynamic environment. Proximal to the carotid bifurcation, the left carotid artery normally experiences laminar flow. After CAL, blood flow in the left carotid artery becomes disturbed, leading to IMT and vascular remodeling in order to normalize shear stress. A main result from the present study is the failure of PECAM knockout mice to assemble FN after CAL. Other studies have focused on the role of haemodynamics in the regulation of expression of ECM components. Atheroprone areas of the vasculature exhibit increased deposition of FN and ECs exposed to disturbed flow exhibit increased FN expression^11^,^35^,^36^,^37^,^38^. Not surprisingly, the role of FN in inflammation and atherosclerosis is rather complex: while FN promotes increased plaque area, it has also been shown to promote formation of the protective fibrous cap, which in humans prevents plaque rupture^39^. Our results are in agreement and extend these findings to show increased FN deposition in areas of disturbed flow in response to CAL. Importantly, PECAM knockout mice, which lack much of their endothelial shear sensing capability, deposit very little FN and exhibit almost no fibril assembly. The observed deficiency in FN fibrillogenesis in the absence of PECAM could be the result of two causes: a reduction in FN expression or a lack of endothelial capacity to physically assemble the fibrils. Our results show that both are the case in PECAM KO ECs. We observe that PECAM KO cells exhibit deficient basal FN mRNA expression, consistent with previous work showing that atheroprone shear stress turns on transcription of FN via a PECAM/NFκB-dependent pathway in ApoE KO mice^36^. We also show that when provided exogenous FN, KO cells are unable to assemble the FN into fibrils, demonstrating an intrinsic inability of KO cells to remodel FN, independent of the expression of FN.

RhoA-mediated contractility is thought to be the physical force that stretches FN proteins and assembles them into fibrils^22^,^32^,^40^. We show here that PECAM is required for RhoA activity in ECs, as PECAM KO ECs display reduced levels of active, GTP-bound RhoA. Importantly, gain-of-function studies show that stimulation of RhoA activity in KO ECs leads to increased FN assembly. Reciprocally, loss-of-function studies using siRNA-mediated knockdown of RhoA in PECAM-expressing ECs show that abrogation of Rho signaling results in significant attenuation of FN assembly. Since β1 integrins are the main integrins that bind to the RGD domain of FN and shear stress leads to increased integrin activation and increased binding to the ECM^41^, we examined the activation of β1 integrins in PECAM^−/−^ carotid arteries. We found that PECAM is required for increased levels of active β1 integrins. A causative role for β1 integrins in FN assembly was shown using two complementary approaches: use of siRNA-mediated knockdown and function blocking antibodies, both of which resulted in reduced RhoA activation and downstream FN assembly. This demonstrates a requirement for β1 integrin in PECAM-mediated FN assembly.

Taken together with previous studies, our results here provide a mechanistic link between endothelial mechanosensing, FN assembly, and flow-mediated vascular remodeling. Although the observation that FN fiber organization shows an apparent flow dependency was made more than 20 years ago, the molecular mechanisms of this process remained a mystery. We now show that shear stress serves as a potent activator of β1 integrins and RhoA signaling through a PECAM-dependent mechanism that, in turn, drives FN (and CL) assembly. Our results show that increased FN assembly resulting from chronic disturbed flow *in vitro* and *in vivo* requires PECAM, indicating that FN assembly is a mechanoresponsive event. Previous work from our lab described a requirement for PECAM in flow-induced inflammation, IMT and vascular remodeling in a model of partial carotid ligation^26^. Using two dfferent models of atherosclerosis (ApoE KO and LDLR KO), previous studies have shown that PECAM exhibits site-specific behavior in the formation and development of atherosclerosis, such that PECAM promotes atherosclerosis in the aortic arch while it prevents atherosclerosis in the descending aorta^42^,^43^,^44^,^45^. Although previous studies have used mice with different genetic backgrounds, dietary regimens and assessed disease at different time points, they all agree that the site-specific differences in PECAM-1 behaviour may be due to differences in haemodynamics between the aortic arch and descending aorta; the former experiences disturbed flow while the latter experiences predominantly laminar flow. The present study shows that under disturbed flow regimes, PECAM promotes FN assembly. Given that FN assembly is required for vascular remodeling and attendant inflammation^2^, and that haemodynamics regulate FN deposition^36^, increased FN assembly may be a hallmark of vessel inflammation caused by PECAM signaling in response to disturbed flow. It is therefore tempting to hypothesize that PECAM- dependent FN assembly underlies inflammatory remodeling, such as IMT and even atherosclerosis, in the vasculature. However, further studies are needed to delineate if FN assembly begets endothelial inflammation, or whether the converse is true and endothelial inflammation begets FN assembly. Further research in this area could lead to targeted therapies to atherosclerotic areas, where plaque progression could be slowed or fibrous caps could be stabilized through mechanisms that block or induce FN assembly, respectively.

## Materials and Methods

### Cell culture, shear stress assays

PECAM^−/−^ (KO) cells and cells reconstituted (RC) with full-length PECAM were prepared as described^46^. Levels of PECAM in reconstituted cells are similar to wild-type levels. Primary bovine aortic endothelial cells (BAEC) were obtained from VEC Technologies (Rensselaer, NY). Nearly confluent cells were incubated in starvation media (DMEM with 0.5% fetal bovine serum and 1% penicillin/streptomycin) overnight before shear stress experiments. Cells on fibronectin-coated glass slides were mounted in a parallel plate flow chamber connected to a NE-1050 bi-directional syringe pump by rigid wall tubing (New Era Pump Systems, Inc., Farmingdale, NY). Cells were exposed to oscillatory flow for 24 hours at ± 6.5 Dynes/cm^2^ with a frequency of 1 Hz. Flowed slides were washed with cold phosphate buffered saline (PBS) for Rho-GTPase pulldown, deoxycholate (DOC) solubility assays or fixed with 1% formaldehyde for 10 min for immunostaining as described below. Cells not subjected to shear stress were used as static controls.

### DOC solubility assay

Cells were washed with cold PBS for 3 times, and lysed in cold 2% DOC buffer^47^. Cell lysates were processed with a bead homogenizer for 30 s and aliquoted. To separate the DOC-insoluble fraction, an aliquot was centrifuged at 16000 rpm in 4 °C for 15 m. The pellet was dissolved in SDS loading buffer and DOC-insoluble proteins were examined with Western blot assay. BCA assays were performed to ensure equal protein loading, and DOC-insoluble vimentin is used as a loading control protein.

### Western Blotting

Samples were loaded on to pre-cast 4–15% polyacrylamide gradient gels (Bio-Rad, Hercules, CA). Using MOPS running buffer, electrophoresis was carried out at 200 V for 30 minutes, followed by transfer to a nitrocellulose membrane. Membranes were blocked for 1 hour in 50% Li-Cor blocking buffer in 1X PBS (Li-Cor, Lincoln, NE). Membranes were then probed with primary antibodies overnight at 4 °C in 50% Li-Cor blocking buffer in 1X PBS. Primary Antibodies: Goat anti-β1 integrin (1:200; Santa Cruz Biotechnology, Dallas, TX; SC-6622), Rabbit anti-Fibronectin (1:1000; Sigma-Aldrich Inc., St. Louis, MO; F3648), Mouse anti-RhoA (1:500; Santa Cruz Biotechnology, Dallas, TX; SC-418), Mouse anti-GAPDH (1:1000; Millipore, Billerica, MA; MAB374), Goat anti-vimentin (1:200; Santa Cruz Biotechnology, Dallas, TX; SC-7559). Membranes were then probed using fluorescent secondary antibodies for 1 hour at room temperature, followed by scanning on a Li-Cor Odyssey scanner. Fluorescent secondary antibodies were all used at a 1:2000 dilution.

### Quantitative PCR

Quantitative PCR was performed using SYBR Green 2X master mix as per manufacturer’s instructions (Thermo Fisher Scientific, Waltham, MA). Primer Sequences: Fibronectin- F:TGGTTTGGTCTGGGATCAAT; R:ACAGTGCTGCAGGTCAGATG. GAPDH- F: CATGTTTGTGATGGGTGTGA, R:CTAAGCAGTTGGTGGTGCAG. Cycler Conditions: 5 minutes at 95 °C, followed by 45 cycles of: 20 seconds at 94 °C, 20 seconds at 60 °C, and 20 seconds at 72 °C. The last step of the cycle was a melt curve to test primer efficiency. qPCR peaks were quantified using the delta-delta Ct method.

### FITC-Fibronectin assay

1 mg/ml fibronectin stock was conjugated to fluorescein isothiocyanate isomer I (Sigma-Aldrich Inc., St. Louis, MO) as per manufacturer instructions. After dialysis, protein concentration was determined using a BCA assay. Slides were then coated with 10 μg/ml FITC-FN or 0.1% gelatin for 1 hour at room temperature. PECAM RC or PECAM KO cells were plated and allowed to grow to confluence, after which they were serum starved for 16 h. After serum starvation, reduced serum media was either replaced with fresh media or fresh media with 10 μg/ml FITC-FN added. Cells were incubated with FITC-FN for 24 h, followed by fixation and mounting with DAPI-containing media.

### Rho-GTPase assay

Cells were plated on fibronectin-coated glass slides and allowed to grow overnight to 95% confluency. Rho-GTPase pulldown was performed with RBD-conjugated beads per manufacturer’s instructions (Cytoskeleton Inc., Denver CO). To stimulate Rho-GTP, lysophosphatidic acid (LPA) (Sigma-Aldrich Inc., St. Louis, MO) was dissolved in deionized water and added to cell media at 20 μM final concentration. Deionized water was added to control cells. To inactivate Rho-GTP, cells were transfected with RhoA siRNA as described below.

### siRNA transfection

The following siRNA duplexes were synthesized (Dharmacon Inc., Lafayette, CO): bovine β1 integrin CUUAAUAUGUGGAGGAAAUUU; bovine RhoA: CUAUGUGGCAGAUAUUGAdTdT BAECs were plated in 100mm dishes to grow to 30–40% confluence in full media (DMEM with 10% FBS and 1% penicillin/streptomycin). 25 μl 20 μM siRNA was mixed with 500 μl 2x HBS pH 7.05, followed by mixing with 30 μl 2.5 M CaCl_2_. The mixture was incubated at room temperature for 20 min to form siRNA-calcium phosphate complex. The siRNA complex was added to the cell media dropwise. The cells were incubated with siRNA complex overnight, trypsinized and re-plated to a fibronectin-coated dish, and culture in full media. Total β1 integrin protein was monitored before, 24, 48 and 72 hours after transfection. Cells were harvested for Rho-GTPase assays or DOC solubility assay 48 hours after the initial transfection.

### Immunofluorescence

Cells subjected to shear stress were washed 3 times with cold PBS, followed by fixation with 1% formaldehyde for 10 min. Cells were permeabilized with 0.3% Trition X-100 in tris-buffered saline (TBS) for 3 m, and blocked with 0.5% non-fat dry milk, 1% BSA and 0.3% Triton X-100 in TBS for 60 m. A primary antibody, fibronectin (1:200; Sigma-Aldrich Inc., St. Louis, MO; F3648), active β1-integrin (clone 9EG7, 1:100, BD, 553715; or clone HUTS4 1:100; Millipore, Billerica, MA; MAB2079Z) or beta-catenin (1:100, Sigma-Aldrich Inc., St. Louis, MO; C7082), was incubated with the cells overnight in 4 °C, followed by the incubation with Alexa 568-labeled goat anti-rabbit or mouse antibodies (1:200, Life Technologies, Grand Island, NY). Images were taken with a Carl-Zeiss 700 laser confocal microscope.

### Carotid artery ligation model

Wild-type C57/BL6 (PECAM^+/+^) mice were purchased from Jackson Laboratories. PECAM^−/−^ C57BL/6 mice were kindly provided by Dr. P. Newman (Blood Research Institute, Blood Center of Wisconsin, Milwaukee), bred in house and used in accordance with the guideline of the National Institute of Health and for the care and use of laboratory animals (approved by the Institutional Animal Care and Use Committees of the University of North Carolina at Chapel Hill). Male PECAM^−/−^ and age-matched littermates (WT, 10–14 weeks) were used for all experiments. Blood flow reduction in the left common carotid artery (LCA) and blood flow measurements were performed as previously described^26^,^48^. The ligation operation closed internal, external and occipital carotid arteries, and left the thyroid artery open. Partial blood flow (5–10% within 3 weeks after surgery) remained in common carotid artery through the opening thyroid branch. The sham procedure consisted of vessel isolation and ligature placement without ligation.

### *En face* preparation of carotid arteries

The common carotid arteries were perfusion-fixed with 1% paraformaldehyde for 10 m and harvested 5 days or 3 weeks after carotid artery ligation. The adventitia was removed using surgical forceps. The remaining intimal-medial tissue of the carotid arteries was cut longitudinally and mounted flat on glass slides for immunofluorescence and imaging.

### *En face* tissue immunofluorescence

*En face* mounted tissue samples were rehydrated with water for 10 minutes. The tissue was then permeabilized using Tris-Triton buffer (50 mM Tris, 500 mM NaCl, 0.3% Triton X-100) for 1 hour, followed by 3 washes with water. Permeabilized tissue was then blocked using 0.5% non-fat dry milk and 1% BSA in TBST for 60 minutes at room temperature. Primary antibodies (Rabbit anti-FN, 1:100, Sigma F3648, St. Louis, MO; rat anti-VE-Cadherin, 1:50, BD Pharmingen 550548, Franklin Lakes, NJ,; or rabbit anti-Collagen α1, 1:100, Abcam Ab292, Cambridge, UK) were then diluted in blocking buffer and incubated with the tissue overnight at 4 °C. After primary antibody incubation, slides were washed 3 times with TBST. Secondary antibodies (Goat anti-rat Alexafluor 488, 1:100, A11006 Life Technologies; Goat anti-rabbit Alexafluor 568, 1:100, A11011 Life Technologies) were diluted in blocking buffer and incubated with the tissue for 60 minutes at room temperature. The tissue was washed 3 times in TBST, and then mounted with VectaShield plus DAPI (Vector Labs, Burlingame, CA) and sealed with clear nail polish. Slides were imaged on a Zeiss 700 or Zeiss 710 confocal microscope.

### Percent Fibril Area Calculations and Fibril Angle Morphometry

Grayscale images of fluorescently labeled FN were opened in Fiji image analysis software. The images were then adjusted using the threshold command with the “dark background” option selected. The threshold values were recorded and used across all images for each experiment. After thresholding, the analyze particles command was used, which produces the area of each fibril in an image. These fibril areas were added together to obtain the total fibril area. This area was then divided by the total image area, giving the percent fibril area. To obtain fibril angles, thresholding was performed, followed by analysis using the Directionality plugin in Fiji. Angles from 90 to −90 were analyzed, and the resulting particle frequencies were grouped together in 10-degree bins, followed by plotting in Microsoft Excel.

### β1 integrin function blocking studies

BAECs were grown to 95% confluence, followed by 4 hours of serum starvation in 0.5% FBS. BAECs were then treated with either 1 μg/ml β1 integrin function blocking antibody AIIB2 (University of Iowa Hybridoma Bank) or 1 μg/ml ChromPure mouse IgG (Jackson Immunoresearch Laboratories, West Grove, PA; 015-000-003) for 24 hours at 37° and 5% CO_2_.

### Statistical Analysis

Results are described as mean ± SEM. Statistical tests were performed with Microsoft Excel analysis package, using Student’s t-test for 2 groups or one-way ANOVA followed by multiple comparisons with Tukey’s Honest Significance Difference test. Level of p < 0.05 was considered significant.

## Additional Information

**How to cite this article:** Chen, Z. *et al*. Haemodynamics Regulate Fibronectin Assembly via PECAM. *Sci. Rep.* **7**, 41223; doi: 10.1038/srep41223 (2017).

**Publisher's note:** Springer Nature remains neutral with regard to jurisdictional claims in published maps and institutional affiliations.

## Supplementary Material

Supplementary Information
