# Supplementary material for: Haemodynamics Regulate Fibronectin Assembly via PECAM
Source: Sci Rep. 2017 Jan 25;7:41223. doi: 10.1038/srep41223 (PMC5264604; doi:10.1038/srep41223)
Supplement: Supplementary Information [file srep41223-s1.pdf]

Haemodynamics regulate fibronectin assembly via PECAM

Zhongming Chen, Chris Givens, John S Reader and Ellie Tzima

Figure S1

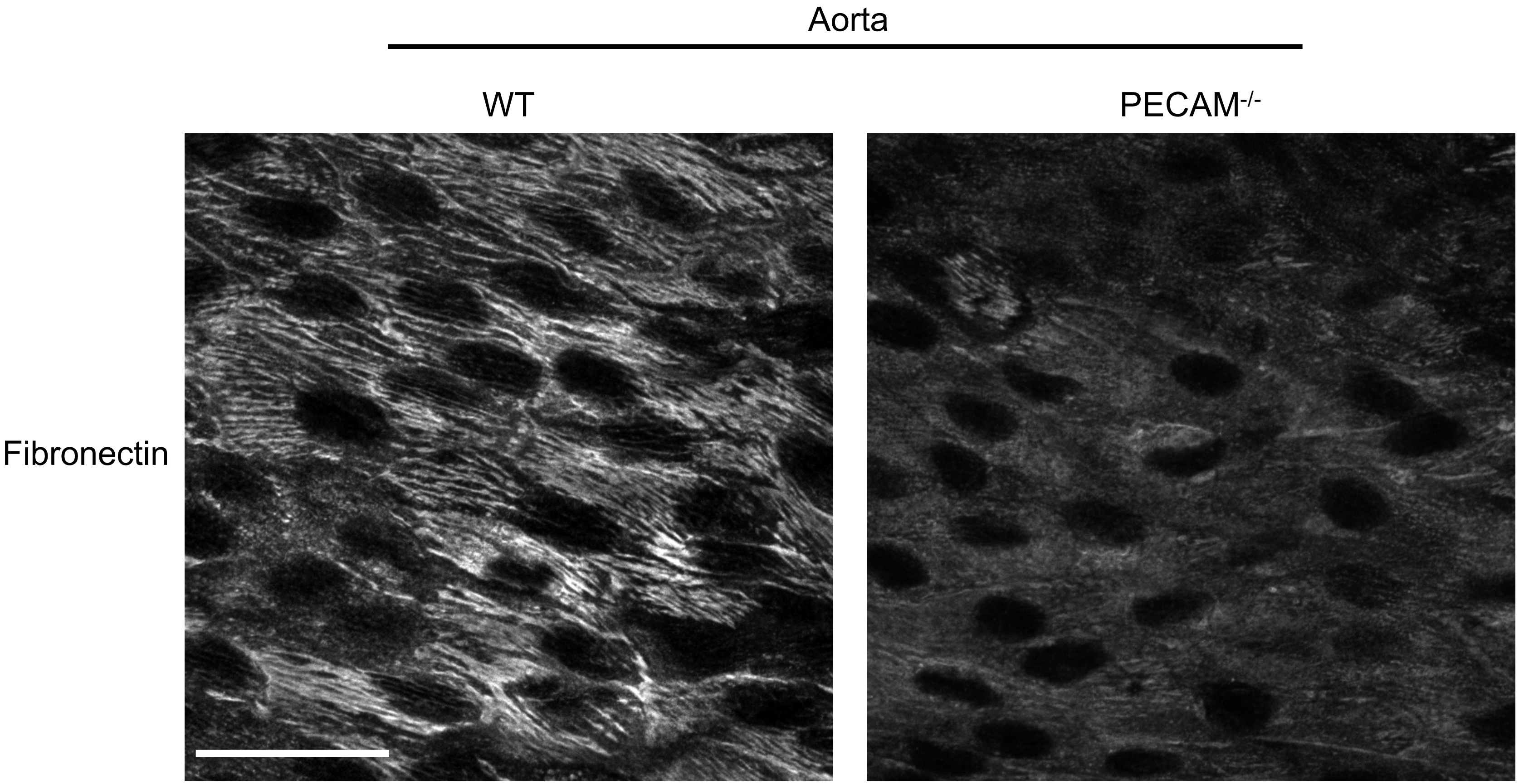

Figure S1: PECAM-1 is required for FN assembly in the descending aorta WT and PECAM$^{-/-}$ aortas were mounted *en fac*e and stained for fibronectin. Scale Bar = 20μm, n=1 mouse per genotype.

Figure S2

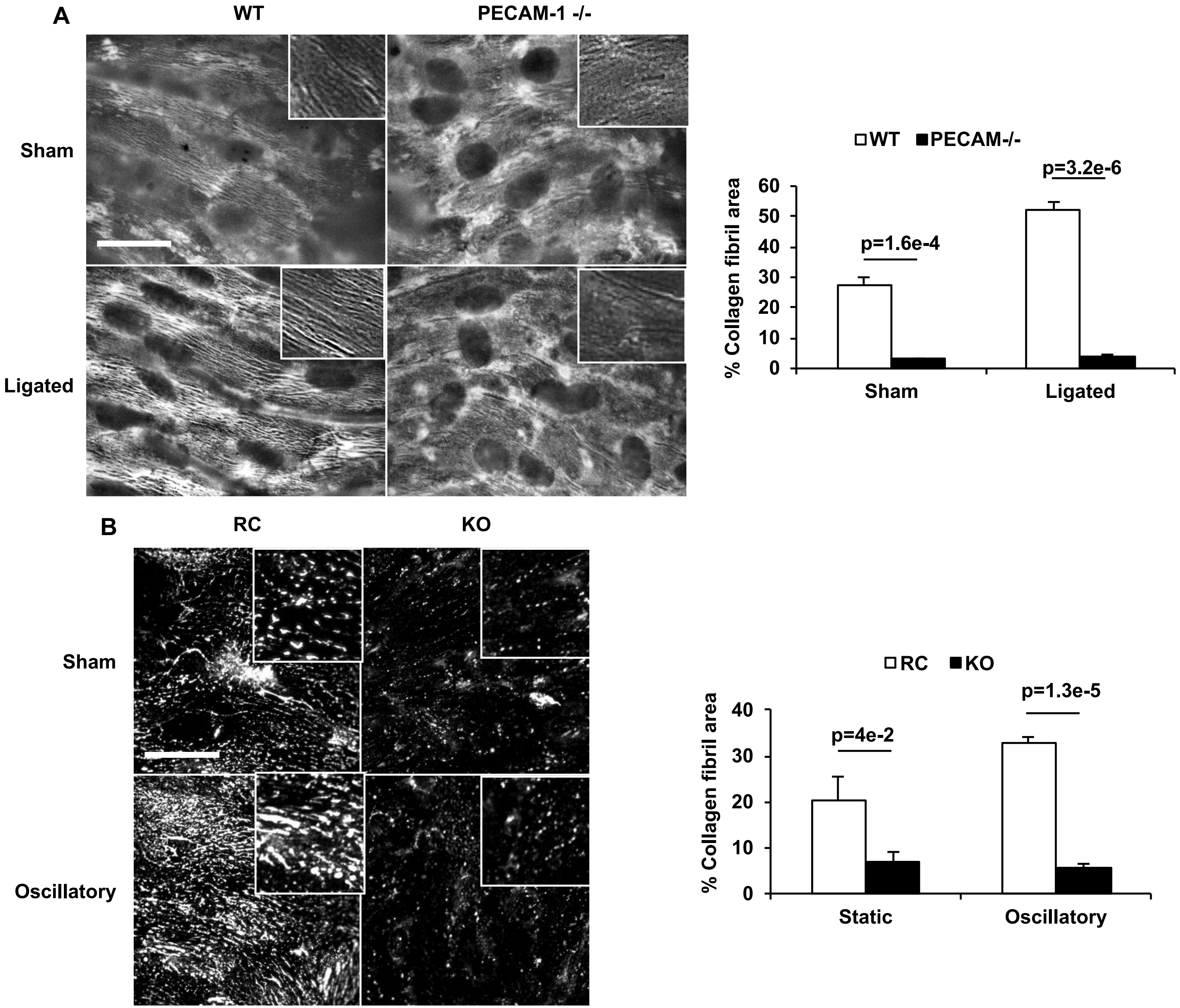
A
WT
PECAM-1 -/-
Sham
Ligated
□WT ■PECAM-/-
% Collagen fibril area
60
50
40
30
20
10
0
p=1.6e-4
p=3.2e-6
Sham
Ligated
B
RC
KO
Sham
Oscillatory
□RC ■KO
% Collagen fibril area
40
30
20
10
0
p=4e-2
p=1.3e-5
Static
Oscillatory

Figure S2: PECAM-1 is required for shear-induced CL assembly (A) WT and PECAM$^{-/-}$ mice were subjected to CAL. 5 days after CAL, sham and ligated carotid arteries were harvested, mounted *en face* and stained for CL. CL fibril area was quantified. Scale bar = 30μm, Sham n = 5 WT and 4 PECAM-1$^{-/-}$, 5d CAL n = 4 WT and 4 KO. (B) RC and KO cells were subjected to flow, followed by immunostaining for CL. CL fibril area was quantified. Scale bar = 10μm, n= 3 independent experiments.

Figure S3

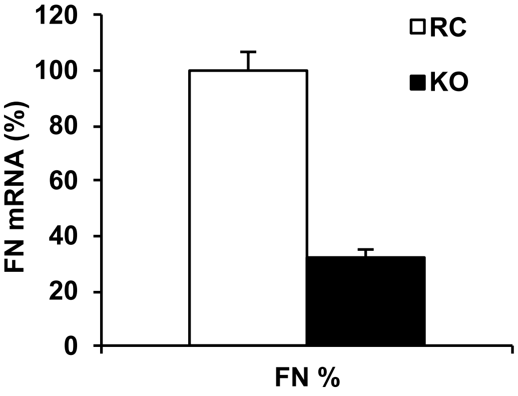

Figure S2: PECAM-1 is required for FN expression mRNA was harvested from RC and KO cells, and qPCR was performed using primers specific to FN. n = 3 biological replicates for each genotype.
